# Brain pericyte biology: from physiopathological mechanisms to potential therapeutic applications in ischemic stroke

**DOI:** 10.3389/fncel.2023.1267785

**Published:** 2023-09-14

**Authors:** Jiaqi Fu, Huazheng Liang, Ping Yuan, Zhenyu Wei, Ping Zhong

**Affiliations:** ^1^School of Health Science and Engineering, University of Shanghai for Science and Technology, Shanghai, China; ^2^Department of Neurology, Shidong Hospital, Yangpu District, Shanghai, China; ^3^Monash Suzhou Research Institute, Suzhou, Jiangsu, China; ^4^Department of Cardio-Pulmonary Circulation, Shanghai Pulmonary Hospital, School of Medicine, Tongji University, Shanghai, China

**Keywords:** brain pericytes, ischemic stroke, stem cell characteristics, contractile characteristics, paracrine characteristics

## Abstract

Pericytes play an indispensable role in various organs and biological processes, such as promoting angiogenesis, regulating microvascular blood flow, and participating in immune responses. Therefore, in this review, we will first introduce the discovery and development of pericytes, identification methods and functional characteristics, then focus on brain pericytes, on the one hand, to summarize the functions of brain pericytes under physiological conditions, mainly discussing from the aspects of stem cell characteristics, contractile characteristics and paracrine characteristics; on the other hand, to summarize the role of brain pericytes under pathological conditions, mainly taking ischemic stroke as an example. Finally, we will discuss and analyze the application and development of pericytes as therapeutic targets, providing the research basis and direction for future microvascular diseases, especially ischemic stroke treatment.

## 1. Introduction

Although Eberth described the existence of pericytes in 1871, pericytes are generally considered to have been discovered by the French scientist Charles-Marie Benjamin Rouget (Eberth, 1871; Rouget, 1873; Armulik et al., 2011). Later, in the 20th century, German scientist Karl Wilhelm Zimmermann named these cells “Rouget cells” and coined the term “pericytes” to indicate their proximity to endothelial cells (Armulik et al., 2011). Pericytes in different organs may differ in morphology, plasticity and function. Even within the same organ, there are different subgroups of pericytes (Birbrair, 2018). For example, in the central nervous system (CNS), pericytes can be divided into three subtypes according to their different morphology and region-specific distribution: sheath-like pericytes, reticular pericytes and thin-chain pericytes.

### 1.1. Distribution of pericytes

Pericytes are mainly distributed around capillaries and microvessels, extending along capillaries, pre-capillary arterioles and post-capillary venules, but are not commonly found in lymphatic capillaries (Armulik et al., 2011; Sweeney et al., 2016; Winkler et al., 2017). They are candidates for regulating microcirculatory blood flow because they strategically cover 90% of the capillary surface area and can respond quickly to neuronal stimuli (Alarcon-Martinez et al., 2020). According to substances enriched in pericytes and their difference in functions, they can be divided into two subtypes: T-pericytes enriched in small molecule transmembrane transporters (for transport) and M-pericytes enriched in extracellular matrix organization (for matrix) (Zhang et al., 2021; Yang et al., 2022). Pericytes in different capillary segments may exhibit different functions. For example, pericytes in the middle segment of capillaries mainly show a spiral or femoral staining pattern. These pericytes do not express isoforms of smooth muscle actin (SMA), whereas transitional pericytes in the pre- and post-capillary segments mainly show a reticular pattern and express α-SMA at higher frequencies and intensities. This indicates that the contractile ability of connecting pericytes is stronger than that of mid-capillary pericytes. Therefore, regulation of capillary blood flow may be accomplished by pre-capillary and post-capillary pericytes (Nehls and Drenckhahn, 1991; Alarcon-Martinez et al., 2021). However, on the arteriovenous axis, the transcriptional gradient within pericytes is independent of their position along it (Garcia et al., 2022). At the same time, there is a wide variation in pericyte density between individuals and within individuals. For example, CNS pericyte density of platelet-derived growth factor receptor β(PDGFRβ) +/− and F7 mice is highest with an endothelial cell/pericyte ratio of 1:1 (Armulik et al., 2010; Bell et al., 2010; Zhang et al., 2020). In contrast, Crouch et al. reported a ratio of 1:2.8 in the subventricular zone and 1:3.7 in the cortex in 12-month-old mice using FACS sorting (Crouch et al., 2015). In particular, when there is a region-specific lack of pericytes, it can lead to vascular instability and promote vascular rupture. For example, when the cell density is <50% of the normal values in retinal microvessels, proliferative retinopathy inevitably occurs (Enge et al., 2002; Santos et al., 2019). In addition, pericyte loss appears to be associated with increased capillary diameter due to endothelial cell proliferation, morphological and ultrastructural abnormalities with changes in the distribution of certain junctional proteins, leading to disruption of the microvascular structure and increased vascular permeability due to increased transendothelial permeability (Hellström et al., 2001).

### 1.2. Biomarkers for pericytes

Previously it was impossible to accurately distinguish between pericytes and other vascular-associated cells because light and electron microscopies were the only technologies capable of visualizing these cells, limiting the information obtained. With the emergence and development of fluorescence microscopy confocal microscopy and genetic tools (such as fate lineage tracing), more molecular markers have been discovered for identifying pericytes, such as PDGFRβ, neuroglial antigen 2 (NG2), melanoma cell adhesion molecule (CD146), etc (Table 1; Armulik et al., 2011; Santos et al., 2019; Alarcon-Martinez et al., 2021; Naranjo et al., 2022). Under normal conditions, PDGFRβ is only expressed in pericytes and is evenly distributed in the hippocampus. After Injury, it accumulates around cortical thalamic injury sites and is also expressed in neural progenitor cells, glial cells, and fibroblasts (Bell et al., 2010; Kyyriäinen et al., 2017). Chondroitin sulfate proteoglycan 4/ NG2 is always expressed by wall cell components of neovascular structures, such as cardiomyocytes in embryonic hearts, developing smooth muscle cells in large vessels, and pericytes in neovascular microvessels (Ozerdem et al., 2001; Stallcup, 2002). CD146 has also been used as a marker for pericytes. In mouse brains, CD146 is first expressed on immature capillary endothelial cells not covered by pericytes. As the pericyte coverage rate increases, CD146 is only expressed in pericytes and is not seen in brain vascular endothelial cells (Chen et al., 2017). Nestin is a type VI intermediate filament generally used for the identification of neural stem cells and research has found that nestin is also positive in pericytes (Klein et al., 2014).

**Table 1:** Pericyte markers (Rouget, 1873; Enge et al., 2002; Bell et al., 2010; Alarcon-Martinez et al., 2021).

Pericyte marker	Gene symbol	Examples of other cell types expressing the marker	Comments
PDGFR-β (platelet-derived growth factor receptor beta)	Pdgfrb	Also expressed by mesenchymal stem cells during development; fibroblast cells during injury; CNS certain neurons and neuronal progenitors; glia; fibroblasts; myofibroblasts; smooth muscle cells	Receptor tyrosine kinase; functionally involved in pericyte recruitment during angiogenesis; useful marker for brain pericytes
NG2 (chondroitin sulfate proteoglycan 4)	Cspg4	Also expressed by developing cartilage, bone, muscle; early postnatal skin; adult skin stem cells; neuronal progenitors; oligodendrocyte progenitors; adipocytes; vSMCs; Schwann cells; perineurial cells	Integral membrane chondroitin sulfate proteoglycan; involved in pericyte recruitment to tumor vasculature
CD13 (alanyl (membrane) aminopeptidase)	Anpep	Also expressed by ECs; inflamed and tumor endothelium; myeloid cells; epithelial cells in the kidney and gut; vSMCs	Type II membrane zinc-dependent metalloprotease; useful marker for brain pericytes
aSMA (alpha-smooth muscle actin)	Acta2	Also expressed by vSMCs; myofibroblasts; myoepithelium	Structural protein; quiescent pericytes do not express aSMA (e.g., CNS); expression in pericytes is commonly upregulated in tumors and in inflammation
Desmin	Des	Also expressed by skeletal, cardiac, smooth muscle	Structural protein; useful pericyte marker outside skeletal muscle and heart
Nestin	Nes	Expressed in a subpopulation of pericytes (Type-2); progenitor cells	
RGS5 (regulator of G protein signaling 5)	Rgs5	Also expressed by cardiomyocytes; vSMCs	Regulate heterotrimeric G proteins by activating GTPase activity; angiogenic pericyte marker
SUR2 (ATP-binding cassette, member 9)	Abcc9	Also expressed by skeletal, cardiac, smooth muscle; renal tubular epithelium	Regulatory subunit of ATP-sensitive potassium channels
Kir6.1 (K^+^ inwardly-rectifying channel, subfamily J, member 8)	Kcnj8	Also expressed by vSMCs	Associates with SUR2
MCAM (melanoma cell adhesion molecule)	CD146	Also expressed in vSMCs; mesenchymal stem cells; immature endothelial cells; ganglion cells; activated T lymphocytes	
Endosialin	Cd248	Also expressed by vSMCs; myofibroblasts; fibroblasts; T cells;	Transmembrane cell surface glycoprotein; expression on pericytes is dynamic; downregulated during development
DLK1 (delta-like 1 homolog)	Dlk1	Also expressed by vSMCs; hepatoblasts in the developing liver; adipocyte progenitors	Transmembrane cell surface protein
Tbx18	TBX18	Also expressed in SMCs	
PDGFRa (platelet-derived growth factor receptor alpha)	Pdgfra	Also expressed in oligodendrocyte precursor cells and fibroblast-like cells	
Glast	SLC1A3	Expressed only in a subpopulation of spinal cord pericytes	
Myh11	MYH11	Labels only arteriolar pericytes	
Leptin receptor	LepR	Labels sinusoidal pericytes	
Transgelin	SM22a	Also expressed in vSMCs	Express lower than vSMC
Calponin1	CNN1	Also expressed in vSMCs	Express lower than vSMC
Vitronectin	Vtn		Be specific to identify pericytes
Interferon-induced transmembrane protein 1	Ifitm1		Be specific to identify pericytes
Gli1	Gli1	Labels several perivascular cells	
XlacZ4		Also expressed in vSMCs; skeletal muscle progenitors during development	Expresses nuclear beta-galactosidase in pericytes and vSMCs; downregulated in association with injury
NG2 dsRED		Also expressed in vSMCs, oligodendrocyte progenitors	
NeuroTrace 500/525		No reported expression	

Fluorescent Nissl dye NeuroTrace 500/525 has recently been found to specifically label brain pericytes achieving high-resolution optical imaging of live mice and demonstrating that capillary pericytes are a group of wall cells with unique morphological, molecular, and functional characteristics (Damisah et al., 2017; Naranjo et al., 2022). Recent work in the field of cerebral vasculature has shown that combinations of biomarkers can be effective for the diagnosis of stroke (Jickling and Sharp, 2015; Steliga et al., 2020). Previous research has suggested that better identification of neurovascular unit (NVU) subtypes may be of greater significance, such as PDGFRβ (+) cells for future involvement in endogenous recovery or autologous cell therapy and many methods have been reviewed for distinguishing NVU subtypes, such as multiple driver Cre systems, lentiviruses, and nanoparticles (Jung et al., 2011; Naranjo et al., 2022). Unfortunately, there is currently no single molecular marker that can be used to definitively and specifically label the entire population of pericytes and all markers currently used are dynamic in expression potentially up- or down-regulated with developmental status, pathological response, *in vitro* culture etc. Therefore, the most advanced pericyte identification relies on well-preserved tissue morphology and a combination of two or more pericyte markers (Armulik et al., 2011).

In summary, pericytes are widely distributed in the body and participate in diverse physiological functions. The origin, development, distribution, and biological markers have been comprehensively reviewed in the literatures, but biological characters of pericytes and their roles in specific neurological disorders have not been thoroughly described. Therefore, the present study searched literatures on pericytes published between 2000 and 2023 in Pubmed and specifically summarized biological characters of pericytes, especially their stem cell, contractability, and paracrine features. Apart from these, roles of pericytes in ischemic stroke and other neurological conditions have also been discussed, aiming to lay the theoretical foundation for the effective and safe treatment of the abovementioned neurological disorders.

## 2. Functional characteristics of pericytes

**Figure 1:**
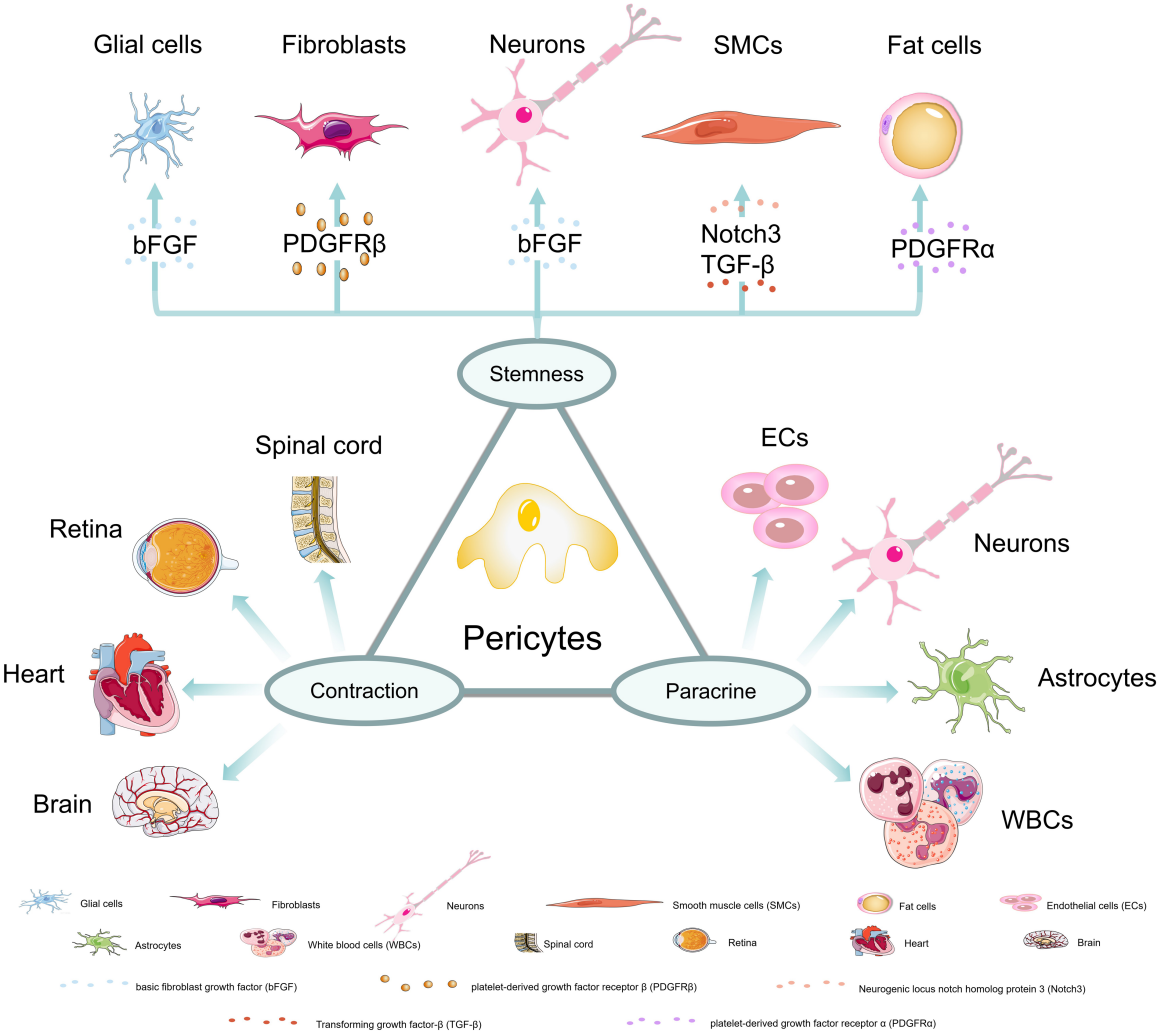
Three major properties of pericytes. (1) stem cell properties: Under the induction of bFGF and other factors, pericytes could differentiate into glial cells, neurons, fibroblasts, smooth muscle cells and fat cells; (2) contractile properties: pericytes in the spinal cord, retina, heart, and brain have contractile abilities; (3) paracrine properties: pericytes can communicate and interact with endothelial cells, neurons, astrocytes and white blood cells to achieve more biological functions.

Different subgroups of pericytes may exist in the same organ and pericytes in different capillary segments may also exhibit different functions. However, overall, pericytes have three main characteristics: stem cell, contractile and paracrine (Figure 1). These characteristics enable pericytes to play an important role in stabilizing the blood–brain barrier, promoting angiogenesis, regulating blood flow, clearing harmful toxins, and regulating immune response (Sweeney et al., 2016; Cao et al., 2021; Guo et al., 2022).

### 2.1. Stem cell characteristics

Studies have shown that pericytes can give rise to adipocytes, cartilage, bone, and muscle (Armulik et al., 2011). For example, Birbrair et al. demonstrated that two subtypes of pericytes in mouse skeletal muscles, Nestin-GFP (−)/NG2-DsRed (+) for type 1 and Nestin-GFP (+)/NG2-DsRed (+) for type 2 pericytes. They promote successful muscle regeneration by balancing myogenic and non-myogenic cell activation. Type 1 pericytes express the adipogenic progenitor marker platelet-derived growth factor receptor α (PDGFRα) and do not form muscles *in vivo* but promote skeletal muscle fat deposition, while type 2 pericytes only promote the formation of new muscles after injury (Birbrair et al., 2013). In the spinal cord microvascular system, PDGFRβ (+) pericytes detach from the vascular wall after spinal cord injury and transform into fibroblasts to form scars (Li et al., 2021). In a mouse model involving developmental fibroblasts, it was also demonstrated that pericytes have the ability to differentiate into fibroblasts and these cells are likely to derive from type A pericytes (Dias et al., 2021). Pericytes also have the ability to differentiate into smooth muscle cells and may be regulated by Notch 3 and transforming growth factor (TGF) -β receptors (Kofler et al., 2015; Bordenave et al., 2020). In addition, adult CNS capillary Nestin (+) NG2(+) pericytes can differentiate into neurons and glial cells under the action of basic fibroblast growth factor (bFGF) (Dore-Duffy et al., 2006). In particular, it has been found that pericytes can transform into microglia with macrophage functions after stroke (Santos et al., 2019). Due to their mesenchymal stem cell characteristics, pericytes have been considered excellent candidates for promoting neural tissue repair and regenerative medicine (Caporarello et al., 2019). In particular, it has been found that glial cell line-derived neurotrophic factor (GDNF) secreted by pericytes can change blood brain barrier (BBB) function and promote brain or peripheral nerve regeneration (Shimizu et al., 2012).

### 2.2. Contractile characteristics

The contractile characteristic of pericytes was first proposed by Rouget (Rouget, 1873). This character is mediated by a unique cytoskeletal organization formed by actin filaments allowing pericytes to deform and transmit mechanical forces to the extracellular matrix to change cell diameter (Sweeney et al., 2016; Nelson et al., 2020; Alarcon-Martinez et al., 2021; Zhu et al., 2022). Nowadays, factors that may affect the contractile characteristic of pericytes have been discovered, such as calcium ions, reactive oxygen, β-adrenergic agonists or antagonists, thromboxane α-2, endothelin-1, etc. (Ahmed and El-Badri, 2018). It has been found that when the spinal cord is in a chronic hypoxic state, it stimulates abnormal overactivation of pericyte monoamine receptors (5-HT1) leading to local contraction of capillaries by pericytes, thereby reducing blood flow to the level of ischemia. In the absence of monoamines, receptor activation is triggered by trace amines (such as serotonin) produced by ectopically expressed aromatic L-amino acid decarboxylase (AADC) in pericytes (Li et al., 2017; Almeida et al., 2018). In the cardiac tissue, pericytes are the second most abundant cell type (Lee and Chintalgattu, 2019; Su et al., 2021). Lee et al. first demonstrated that NG2(+)PDGFRβ(+)CD146(+)CD34(−)CD31(−)CD45(−) cardiac pericytes express contractile proteins and exhibit some contraction and relaxation under adrenergic signaling stimulation in the isolated state (Lee et al., 2021). When myocardial ischemia/reperfusion injury occurs, pericyte contraction is aggravated leading to reduced microvascular reflow (Li et al., 2022). When capillaries are congested in vascular stenosis, pericyte contraction was also observed (Methner et al., 2019). In retinal capillaries, pericytes can regulate capillary diameter according to vascular contractility activity and transmit vascular contractility signals to neighboring pericytes along capillaries (Yao et al., 2022; Kamiya et al., 2023).

### 2.3. Paracrine characteristics

The paracrine characteristics of pericytes support their participation in diverse functions. Currently, it has been found that skeletal muscles and cardiac pericytes play an increasingly important role in maintaining normal tissue homeostasis. In case of muscle injury, pericytes promote regeneration microenvironment by releasing nutritive factors and regulating local immune response (Murray et al., 2017). In the retina, pericytes promote lymphocyte activation by communicating with immune cells and recruiting leukocytes to participate in the clearance of toxic cell byproducts. In the neurovascular unit, they cooperate with astrocytes, neurons, and endothelial cells to regulate and maintain the integrity of the blood–brain barrier (Santos et al., 2019).

## 3. Brain vascular pericytes

So far, the most numerous cell types in the brain are neurons and glial cells, and brain vascular cells account for only 8% of brain cells (Garcia et al., 2022). Although brain vascular cells account for a small proportion, they play an essential role. In recent studies, 14 subtypes of brain vascular cells have been reported including: (a) 3 endothelial cell subtypes separated along the arteriovenous axis (*n* = 2,552); (b) 4 wall cell subtypes (*n* = 1,982), including 1,564 pericytes and 418 smooth muscle cells (SMCs); (c) 3 perivascular fibroblast subtypes with different marker genes (*n* = 793); (d) 4 vascular-coupled (vc) neuron and glial cell subtypes (*n* = 1,520), including vc-neurons, vc-oligodendrocytes, vc-astrocytes and vc-microglia (Garcia et al., 2022). A number of subgroups of pericytes have also been found in cortical vascular structures, including vsmc-pericytes on pre-capillary arterioles, thin-chain spiral pericytes in the middle segment of capillaries, and reticular pericytes with stellate morphology on post-capillary venules (Sweeney et al., 2016; Alarcon-Martinez et al., 2021).

Brain pericytes are an important group of cells located centrally between endothelial cells, astrocytes and neurons within the NVU, with highly heterogeneous origins (Sweeney et al., 2016). Studies have found that forebrain pericytes mainly originate from neural crest cells, while pericytes in other parts of the brain appear to originate from mesodermal mesenchymal stem cells. Some have also proposed that brain pericytes originate from yolk sac-derived macrophage progenitor cells (Armulik et al., 2011; Trost et al., 2016; Zheng et al., 2020; Naranjo et al., 2022). Yamazaki et al. demonstrated that pericytes may originate from tissue marrow progenitor cells (Yamazaki et al., 2017). Yamamoto et al. used a number of transgenic mouse models to reveal that during vascular development brain pericyte subgroups may also originate from phagocytic macrophages (Yamamoto et al., 2017).

Brain pericytes are a morphologically diverse group of neural cells ranging from round smooth muscle-like to elongated fibroblast-like cells (Nehls and Drenckhahn, 1991; Berthiaume et al., 2018). Under electron microscope, pericytes usually protrude forming an oval protrusion surrounded by a number of large protrusions (primary or branching) and secondary protrusions from primary protrusions. The protrusions between adjacent protrusions are very close but do not overlap. The primary protrusions gradually branch and thin to form long pointed ends generally reaching hundreds of micrometers in length forming a partially discrete area occupied by a single pericyte cell chain encircling the capillary (Bergers and Song, 2005; Su et al., 2019; Alarcon-Martinez et al., 2021). In addition to primary and secondary protrusions, some pericytes also show tubular protrusions connecting to neighboring capillary pericytes forming inter-pericyte tunneling nanotubes (Alarcon-Martinez et al., 2021). Using *in vivo* two-photon imaging, Berthiaume et al. found that pericyte protrusions of the adult mouse cortex were inactive but their tips extended and/or contracted over a few days. Selective ablation of individual pericytes led to vigorous extension of the tips of neighboring pericytes toward uncovered endothelial areas (Berthiaume et al., 2018). Brain pericytes are important integrators, coordinators, and effectors of many neurovascular functions, including angiogenesis, stabilization of the blood–brain barrier formation and maintenance, capillary blood flow regulation and toxic cell byproduct clearance, which are essential for CNS homeostasis and normal neural functions (Winkler et al., 2011; Santos et al., 2019; Zhang et al., 2021). Below we will elaborate on the functional role of brain pericytes under normal physiological conditions from the three aspects of stem cell, contractile, and paracrine characteristics.

### 3.1. Stem cell characteristics of brain pericytes

The stem cell characteristics of brain pericytes have been confirmed in many studies, but most studies have shown that pericytes need to be stimulated by certain external factors to induce their differentiation and that pericytes with different cell markers have different differentiation abilities. For example, under the stimulation of bFGF, Nestin (+)NG2(+) αSMA(+) pericytes form small clusters and multicellular spheres and differentiate to express neuronal and glial cell markers, eventually fully differentiating into neural lineage cells (Dore-Duffy et al., 2006). Wojcinski et al. also found similar differentiation phenomena after perinatal ablation of the external granular layer using *in vivo* Cre/loxP technology (Wojcinski et al., 2017). Similarly, Karow et al. also confirmed the above conclusion. By co-expressing the transcription factors Sox2 and Mash1 mediated by a retrovirus, it was demonstrated that brain pericytes can be reprogrammed into neuronal cells (Karow et al., 2012). Under ischemic/hypoxic conditions pericytes can also acquire multipotent stem cell activities and differentiate into major components of the BBB/neurovascular unit (Nakagomi et al., 2015).

### 3.2. Contractile characteristics of brain pericytes

**Figure 2:**
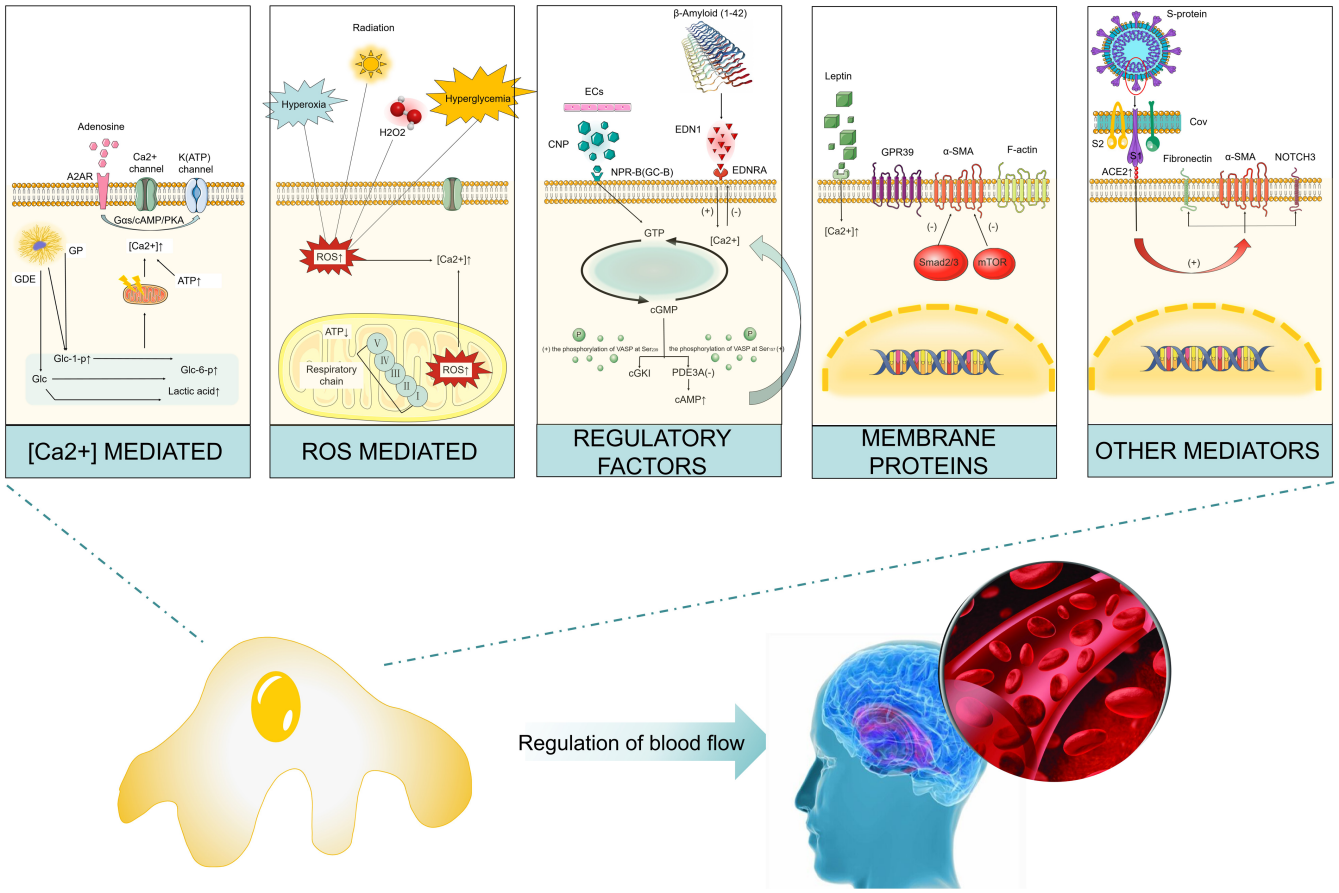
Factors that impact the contractile characteristics of cerebrovascular pericytes. (1) Ca^2+^: the increase in the rate of glycogenolysis decomposition and ATP production leads to the increase in the concentration of intracellular calcium ion and subsequent pericyte contraction; (2) reactive oxygen species: hypoxia, hyperglycemia, hydrogen peroxide, and radiation can result in the increase in intracellular reactive oxygen species, through which the concentration of calcium ion is increased and subsequently pericytes contract; (3) regulatory factors: endothelin-1 induces contraction by acting on its type-A receptor, while CNP can inhibit endothelin-1-induced contraction through the GC-B/cGMP signaling pathway; (4) membrane proteins: inhibition of α-smooth muscle actin expression could prevent contraction, GPR39 knockout results in pericyte contraction; (5) other mediators: SARS virus infection induces pericyte contraction by interacting with cell surface membrane proteins. Abbreviated specification: A2AR-A2A receptors; GP: glycogen phosphorylase; GDE: glycogen debranching enzyme; Glc-1-p: glucose 1- (dihydrogen phosphate); Glc-6-p: glucose 6- (dihydrogen phosphate); Glc: Glucose; ROS: reactive oxygen species; CNP: C-type natriuretic peptide; NPR-B(GC-B): natriuretic peptide receptor-B (Guanylate cyclase B); EDNT: endothelin-1; EDNRA: endothelin Receptor Type A; GTP: Guanosine triphosphate; cGMP: cyclic guanosine monophosphate; cGKI: cGMP-dependent protein kinase I; PDE3A: Phosphodiesterase 3A; cAMP: Cyclic Adenosine Monophosphate; GPR39: G protein-coupled receptor 39; α-SMA: Alpha-smooth muscle actin; mTOR: Mammalian target of rapamycin; ACE2: Angiotensin-Converting Enzyme 2.

Brain pericytes express a variety of contractile proteins (e.g., filamentous actin (F-actin), α-SMA, vimentin, desmin, myosin, nestin) and cytoskeletal proteins (e.g., NG2, PDGFRβ, aminopeptidases A and N (CD13), regulator of G-protein signaling-5 (RGS5), CD146). An increase in the ratio of F- to α-actin not only strengthens the submembrane actin lattice but also promotes actin cross-bridging by extending the length of α-SMA filaments transmitting the generated contractile force to the extracellular matrix to contract blood vessels (Attwell et al., 2016; Sweeney et al., 2016; Alarcon-Martinez et al., 2021). Subsequent studies have also revealed that the contractile characteristics of pericytes may be regulated by a variety of factors, such as calcium ions (Ca^2+^), reactive oxygen species, regulatory factors, and cellular proteins. We will also discuss the contractile characteristics of brain pericytes from these aspects (Figure 2).

#### 3.2.1. Calcium ions

The rhythmic release-replenishment cycle of Ca^2+^ is closely related to the rhythmic contraction of blood vessels (Alarcon-Martinez et al., 2019, 2021). Ca^2+^ signaling activation and conductance activation of K^+^ [IK(Ca) and SK(Ca)] channels cause vascular dilation and induce endothelial cell membrane surface hyperpolarization. This hyperpolarization is conducted along endothelial cells through homocellular gap junctions and transmitted to overlying SMCs and pericytes through heterocellular gap junctions. Then this hyperpolarization affects the contraction of smooth muscle or pericytes by controlling the activity of voltage-gated calcium ion channels (Jackson, 2022). Studies have found that ischemia/hypoxia rapidly causes pericyte relaxation which helps to dilate blocked blood vessels and restore cerebral blood flow (CBF). After a period of ischemia, pericytes contract due to a reduced energy supply and may subsequently die in a contracted state leading to capillary contraction (Hall et al., 2014). The ultrastructural characteristic of pericytes is the presence of large mitochondria which may provide energy to regulate intracellular calcium concentration and fuel contraction. It has been shown that when the glycogenolysis rate increases, accumulation of glucose-1-phosphate, glucose-6-phosphate and lactate increases and cellular load increases mitochondrial dysfunction, leading to increased intracellular calcium concentration, resulting in sustained microvascular contraction (Alarcon-Martinez et al., 2019, 2021). In addition, ATP increase has also been shown to cause an increase in cytoplasmic calcium in brain capillary pericytes under ischemic conditions, leading to capillary contraction but ATP-induced contraction is reversible and can be eliminated by intracellular calcium chelation (Hørlyck et al., 2021).

The membrane potential of vascular cells is the physiological bridge that converts brain activity into vascular function. ATP-sensitive K^+^ (K(ATP)) channels are present in both vascular smooth muscles and dense capillary networks composed of capillary endothelial cells and pericytes. This ion channel can robustly control the membrane potential of vascular cells (Sancho et al., 2022). When functional loss or gain-of-function mutations occur in K(ATP) channel coding genes Potassium Inwardly Rectifying Channel Subfamily J Member 8 (KCNJ8) and ATP Binding Cassette Subfamily C Member 9 (ABCC9), it leads to abnormal differentiation of wall cell progenitors into arterial/arteriolar vascular smooth muscle cells resulting in a series of human CNS diseases (Ando et al., 2022). It has been found that adenosine can activate K(ATP) channels through A2A receptors and the Gαs/cAMP/PKA pathway causing pericyte relaxation, dilating capillaries and increasing CBF (Neuhaus et al., 2017; Sancho et al., 2022). In addition, Guo et al. found that itraconazole can promote K(ATP) channel opening by inhibiting the formation of SUR2/EPAC1 complex, reducing calcium influx into pericytes, thereby significantly inhibiting pericyte contraction reducing the number of blocked capillaries and promoting recovery of cerebral blood flow after cerebral ischemia reperfusion (Guo et al., 2022).

#### 3.2.2. Reactive oxygen species

It has been reported that hypoxia can cause vascular smooth muscle cell contraction by producing reactive oxygen, endothelin-1, and 20-HETE, leading to increased intracellular calcium levels in pericytes and significant capillary contraction. However, Hirunpattarasilp et al. (2022) found that increased cytoplasmic and mitochondrial reactive oxygen species (ROS) and endothelin release was not related to pericyte-mediated capillary contraction. Under high glucose treatment conditions, increased ROS and mitochondrial superoxide decreased mitochondrial respiration, ATP production, as well as pericyte contractility (Liu et al., 2021). Some studies have also proposed that hydrogen peroxide and radiation induce reactive oxygen production, leading to contraction of cultured pericytes (Wang et al., 2007; Nishimura et al., 2016; Hu et al., 2017). Therefore, the effect of ROS on the contractile characteristic of pericytes is still controversial and requires more rigorous research to clarify.

#### 3.2.3. Regulatory factors

Mechanistically, a variety of mediators, such as endothelin-1, PDGFβ, nitroprusside and NO have been identified as potential regulatory factors affecting pericyte contractility (Arimura et al., 2012; Hall et al., 2014; Neuhaus et al., 2017). In the cortex of AD brains, amyloid-β promotes an increase in endothelin-1 which subsequently causes transient dose-dependent pericyte contraction mainly through the A-type receptor of endothelin-1 (Alcendor, 2020; Hibbs et al., 2021). Endothelial C-type natriuretic peptide (CNP) activates downstream targets, such as cytoskeletal-associated phosphoproteins, through the GC-B/cGMP signaling pathway and inhibits phosphodiesterases, thereby, increasing the level of cAMP in pericytes and ultimately preventing endothelin-induced pericyte contraction (Špiranec et al., 2018). PDGFβ and nitroprusside are both regulatory factors that cause pericyte relaxation (Arimura et al., 2012; Neuhaus et al., 2017). Pericytes can also dilate and contract in response to some vasoactive signals (such as nitric oxide, phenylephrine, and adenosine) and regulate the blood flow through changing the diameter of capillaries (Tomanek, 2022).

#### 3.2.4. Cellular or membrane proteins

α-SMA and F-actin have both been shown to be involved in pericyte contraction and blocking α-SMA expression with RNA interference or attenuating α-SMA expression with combined inhibition of Smad2/3 and mTOR can prevent pericyte contraction (Alarcon-Martinez et al., 2019, 2021; Kureli et al., 2020; Zhao et al., 2022). G protein-coupled receptor (GPR39) is also closely related to pericyte contraction. Studies have found that GPR39 deficiency exacerbates capillary blood flow after focal cerebral ischemia, leading to aggravated brain injury, microvascular perfusion and neurological functions after experimental stroke (Methner et al., 2021; Xu et al., 2022). In addition, the leptin receptor can also induce pericyte contraction and vascular contraction by inducing a sustained increase in intracellular calcium ions under the action of leptin (Butiaeva et al., 2021).

#### 3.2.5. Other factors

Coronavirus disease 19 (COVID-19) is a respiratory disease caused by severe acute respiratory syndrome coronavirus 2 (SARS-CoV-2). SARS-CoV-2 infects host cells by binding to the transmembrane receptor angiotensin converting enzyme 2 (ACE2) through the viral Spike (S) protein. Studies have found that ACE2 expression in human brain vascular pericytes increases after they are exposed to the S protein, which is accompanied by enhanced expression of contractile and myofibrogenic proteins, such as α-SMA, fibronectin, type I collagen and neurogenic locus notch homolog protein-3 (NOTCH3). The enhanced expression of these proteins causes profound changes in pericyte phenotype and is associated with cell contractile morphology (Khaddaj-Mallat et al., 2021). As mentioned above, changes in intracellular energy can cause cell contraction and exogenous energy stimulation can also induce pericyte contraction. It has been found that light stimulation activates channelrhodopsin receptor 2 (ChR2) leading to pericyte and capillary contraction and reducing capillary diameter by about 8% (Nelson et al., 2020). Observation by two-photon microscopy found that optogenetic stimulation of brain capillary pericytes reduces lumen diameter and blood flow, which is inhibited by a rho kinase inhibitor fasudil (Hartmann et al., 2021).

### 3.3. Paracrine characteristics

**Figure 3:**
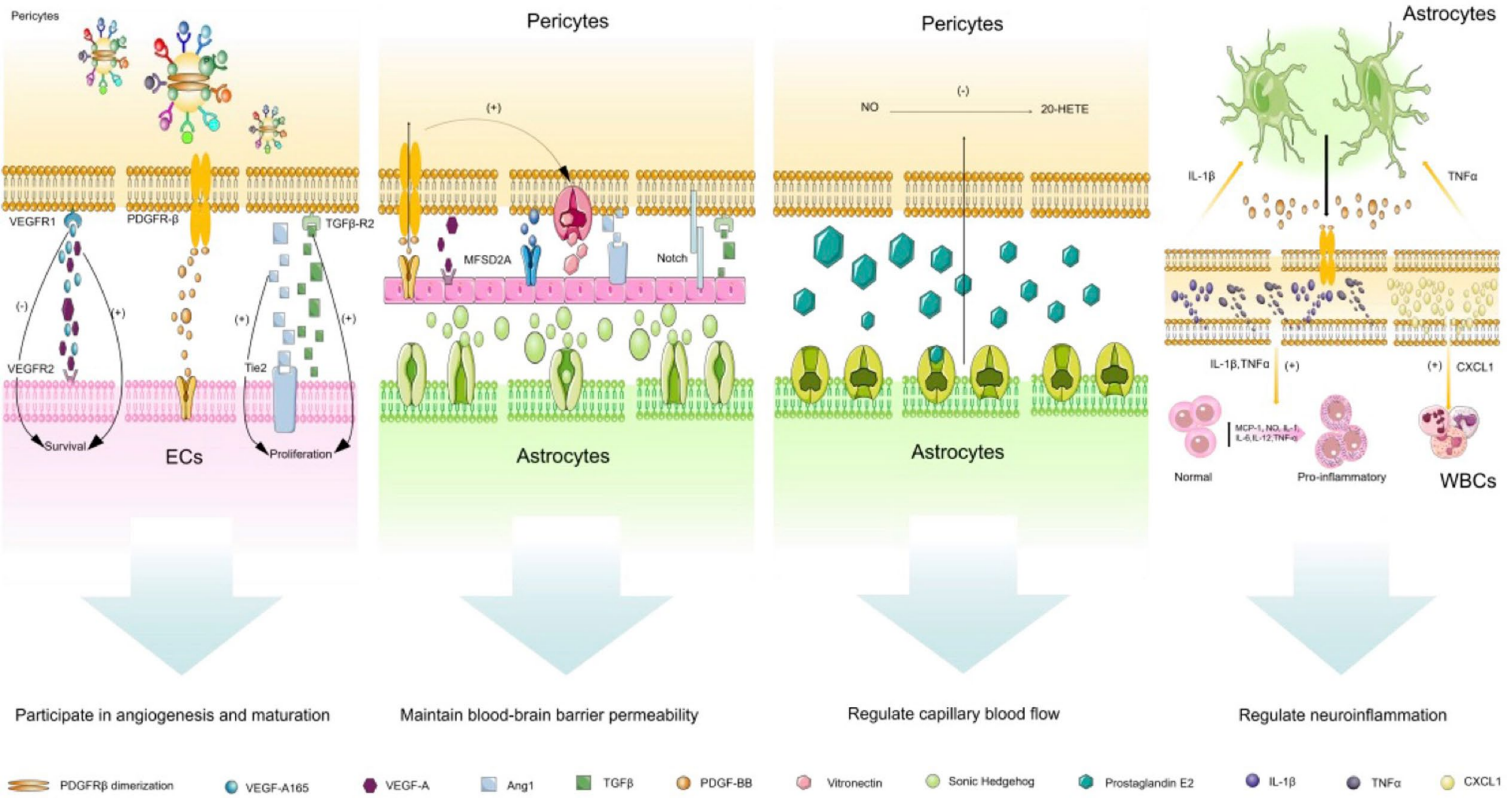
Paracrine characteristics of cerebrovascular pericytes. (1) participation in angiogenesis and maturation: this biological process is mainly due to the communication between pericytes and endothelial cells. VEGF/VEGFR1 pathway is mainly involved in vascular budding, PDGF-BB-PDGFR-β pathway is mainly involved in vascular stability, and TGF-β pathway is mainly involved in vascular maturation; (2) maintenance of the blood–brain barrier permeability: Ang/Tie2 and MFSD2A pathways play a crucial role in regulating the vascular permeability, Sonic Hedgehog from astrocytes can also stabilize the permeability of the blood–brain barrier by acting on endothelial cells; (3) regulation of capillary blood flow: prostaglandin E2 released by astrocytes can actively relax pericytes, but this process requires the release of NO to inhibit the synthesis of 20-HETE which constricts blood vessels; (4) regulation of neuroinflammation: this process mainly involves the communication between endothelial cells, pericytes and astrocytes. Pericytes promote the activation of astrocytes and promote the expression of pro-inflammatory factors in endothelial cells by secreting IL-1β and TNF-α, and pericytes also can release CXCL1 on white blood cells. Abbreviated specification: VEGFA-vascular endothelial growth factor A; VEGFR1: Vascular endothelial growth factor receptor 1; VEGFR2: Vascular endothelial growth factor receptor 2; VEGF-A165:vascular endothelial growth factor A165; PDGFRβ: Platelet-derived growth factor receptor-β; PDGF-BB: Platelet-Derived Growth Factor BB; TGFβ: Transforming growth factor beta; TGFβ-R2: Transforming growth factor beta, type II receptor; Ang1: angiopoietin-1; Tie2: TEK receptor tyrosine kinase; MFSD2A: major facilitator superfamily domain-containing protein 2a; 20-HETE: 20-hydroxy Arachidonic Acid; IL-1β: interleukin-1β; IL-1: interleukin-1; IL-6: interleukin-6; IL-12: interleukin-12; TNFα: tumor necrosis factor α; CXCL1: Recombinant Human C-X-C Motif Chemokine 1; MCP-1: Monocyte Chemotactic Protein 1.

In the brain, pericytes actively communicate with neurovascular unit cells and make fine adjustments in response to stress stimuli (Dore-Duffy, 2008). They integrate, coordinate, and process signals from neighboring cells to produce a variety of functional responses that are essential for the function of the central nervous system in healthy and diseased conditions, including participating in angiogenesis and maturation, maintaining the blood–brain barrier permeability, regulating capillary blood flow, clearing toxic metabolites, and participating in the regulation of neuroinflammation (Sweeney et al., 2016; Naranjo et al., 2022; Figure 3). In the following section, we will discuss the paracrine characteristics of brain pericytes.

#### 3.3.1. Participation in angiogenesis and maturation

During CNS development and vascular remodeling, pericytes can regulate vascular budding, vascular stabilization, and vascular maturation (Sweeney et al., 2016). The process of vascular development and maturation can be specifically divided into the following stages: (1) cell proliferation and migration: pericytes and endothelial cells secrete matrix metalloproteinases (MMPs) to degrade the basement membrane allowing pericytes to detach and endothelial cells to migrate with pericytes transitioning from a quiescent phenotype to an active proliferative phenotype; (2) vascular budding: pericytes regulate migration of endothelial cells through expressing vascular endothelial growth factor (VEGF) promoting vascular budding; (3) vascular stabilization: when endothelial cells form two adjacent buds that fuse in a non-vascular area, endothelial cells recruit more pericytes to stabilize these vessels through PDGF-BB-PDGFR-β signaling in pericytes; (4) vascular maturation: this is achieved through secreting TGFβ and angiopoietin 1 (ANG1) by endothelial cells and pericytes (Payne et al., 2019; Dabravolski et al., 2022).

During the process of vascular budding, pericytes (paracrine signaling) and endothelial cells (autocrine signaling) secrete VEGF-A activating the VEGFR2 pathway on endothelial cells promoting endothelial cell migration, thereby promoting vascular budding (Franco et al., 2011). Local release of VEGF is important for promoting endothelial cell survival and stabilizing new blood vessels (Darland et al., 2003). However, when more soluble homologous VEGF-A165 is released, VEGF does not promote endothelial cell migration, instead, it prevents angiogenesis of mature quiescent vessels due to the presence of VEGFR-1 on the surface of pericytes (Eilken et al., 2017; Caporarello et al., 2019). During the process of vascular stabilization, binding of PDGF-BB secreted by endothelial cells to PDGFR-β ligand on pericytes leads to PDGFR-β dimerization and autophosphorylation of cytoplasmic tyrosine residues. These phosphorylated tyrosine residues become binding sites for proteins containing the SH2 domain, including SFK, PI3K, Shc, RasGAP, STATs, Grb2, Grb7, SH2-containing tyrosine phosphatase (SHP-2 also known as SH-PTP2) and Nck. PDGFR-β controls pericyte proliferation, migration, and recruitment by activating these protein-associated signaling pathways (Tallquist et al., 2003). In the CNS, lack of PDGFR-β leads to reduced pericyte coverage, resulting in endothelial cell proliferation and microvascular instability (Bell et al., 2010; Armulik et al., 2011; Alarcon-Martinez et al., 2021). During the process of vascular maturation, TGF-β/TGF-βr2 and Ang1/Tie2 signaling pathways also play a crucial role. TGF-β mediates proliferation, differentiation, and survival of pericytes and endothelial cells, whereas overexpression of Ang-1 leads to increased restructuring from immature to mature vessels (Uemura et al., 2002; Alarcon-Martinez et al., 2021). It has been found that integrin αVβ8 inhibits TGFβ, thereby inhibiting endothelial cell proliferation and migration. When αVβ8 is added or downstream TGFβ1-TGFBR2-ALK5-Smad3 signaling pathway is missing in the brain, vascular budding, branching, and proliferation is increased, leading to abnormal vascular development and hemorrhage (Arnold et al., 2014).

#### 3.3.2. Maintaining the blood–brain barrier permeability

Pericytes can control the expression of tight and adherens junction proteins, the arrangement of tight junction proteins and vesicle endocytosis at the blood–brain barrier, most of which depend on molecular pathways between endothelial cells and pericytes (Sweeney et al., 2016). As summarized earlier, the main pathways between pericytes and endothelial cells are: the PDGF-BB/ PDGFRβ pathway, TGF-β/TGFβR2 pathway, Notch pathway, VEGF-A/VEGFR2 pathway, Ang/Tie2 pathway, major facilitator superfamily domain containing 2 (MFSD2A) pathway, and the Ephrin/Eph pathway with the Ang/Tie2 and MFSD2A pathways playing a crucial role in regulating vascular permeability (Armulik et al., 2011; Sweeney et al., 2016). In addition, endothelial cells also rely on vitronectin and angiogenin I from pericytes and Sonic Hedgehog from astrocytes to maintain their survival and blood–brain barrier stability. In return, endothelial cells secrete PDGF-BB providing reciprocal survival signals to pericytes through protein kinase B and further stimulating the production of vitreous adhesion protein (Naranjo et al., 2022).

#### 3.3.3. Regulating capillary blood flow

With the development of imaging technology, pericytes have been observed to change the capillary diameter through contraction and to regulate microcirculatory blood flow during functional hyperemia in *in vivo* and *ex vivo* organ culture situations (Armulik et al., 2011; Alarcon-Martinez et al., 2021). In the adult brain, pericytes have also been observed to regulate the capillary diameter by contracting the vessel wall (Bell et al., 2010). Studies have found that prostaglandin E2 released by astrocytes leads to capillary dilation by actively relaxing pericytes, but this process requires the release of nitric oxide to inhibit the synthesis of 20-HETE which contracts blood vessels (Hall et al., 2014; Caporarello et al., 2019). In addition, high levels of carbon dioxide and adrenergic receptors (β-2) expressed by pericytes themselves also mediate pericyte relaxation, thereby achieving fine-tuning of blood flow (Rucker et al., 2000; Caporarello et al., 2019).

#### 3.3.4. Participating in the regulation of neuroinflammation

Pericytes have long been thought to have immune functions as they are able to sense systemic inflammation and secrete chemokines to alter brain functions and activate local microglia (Kaul et al., 2020). In genetically modified mice, increased PDGFRβ signaling has been shown to induce the expression of a series of immune response related genes in pericytes (Olson and Soriano, 2011) and the influence of astrocytes on pericyte behavior is achieved through direct communication of PDGF-BB signaling (Santos et al., 2019). PDGFRβ signaling in pericytes also mediates the proinflammatory response of the blood–brain barrier through transcriptional regulation of a number of chemokines, such as monocyte chemoattractant protein-1 (MCP-1), nitric oxide (NO), interleukins-1 (IL-1), IL-6, IL-12, and tumor necrosis factor-α (TNF-α) in endothelial cells, which leads to macrophage and leukocyte transgression into the brain via blood vessels (Sweeney et al., 2016). Intercellular adhesion molecule-1 expressed by pericytes has been shown to guide leukocytes into tissues through “gates” between pericytes by interacting with integrins on leukocytes (Alarcon-Martinez et al., 2021). The “gates” between pericytes are due to regions of low matrix protein deposition (low expression regions (LERs)) directly aligned with gaps between adjacent pericytes and LERs acting as migration “gates” in all inflammatory responses studied. In the meantime, these regions enlarge under IL-1β stimulation and Chemokine (C-X-C motif) ligand 1 (CXCL1) secreted by pericytes drives crawling of neutrophils and other leukocytes, allowing more inflammatory cells to penetrate blood vessels (Wang et al., 2006; Voisin et al., 2010; Navarro et al., 2016). *In vitro* studies have found that lipopolysaccharide (LPS) can induce mouse brain microvascular pericytes to release NO, IL-12, IL-13, IL-9, IL-10, granulocyte colony-stimulating factor, granulocyte-macrophage colony-stimulating factor, eosinophil chemotactic factor (eotaxin), chemokine ligand (CCL)-3, and CCL-4 among other cytokines (Kovac et al., 2011; Sweeney et al., 2016). Pericytes themselves also express some pro-inflammatory markers, such as IL-1β and TNF-α, which can induce an inflammatory state in astrocytes, microglia, and endothelial cells (O’Carroll et al., 2015; Rudziak et al., 2019). Pericytes also express a large number of macrophage markers and possess macrophage functions. They can clear tissue debris and exogenous proteins injected systemically and/or locally into the CNS and participate in the clearance of amyloid-β (Aβ) toxin in Alzheimer’s disease (Sweeney et al., 2016; Kittikulsuth et al., 2021).

## 4. Brain vascular pericytes and ischemic stroke

Previous studies have shown that pericyte degeneration plays an important role in neurological diseases, such as Alzheimer’s disease (AD), amyotrophic lateral sclerosis (ALS) and type 2 diabetes mellitus (T2DM)-associated microvascular and retinal lesions. Pericyte dysfunction is also associated with human immunodeficiency virus (HIV)-associated dementia, epilepsy, cerebral autosomal dominant arteriopathy with subcortical infarcts and leukoencephalopathy (CADASIL) with subcortical infarcts and leukoencephalopathy, and brain cancer (Sweeney et al., 2016; Santos et al., 2019). It has been found that pericytes mainly mediate brain vascular injury through two parallel pathways: (1) reduced cerebral microcirculation leading to reduced brain capillary perfusion, reduced cerebral blood flow, ultimately leading to chronic perfusion stress and hypoxia; (2) blood–brain barrier disruption, leading to secondary neurodegenerative changes (Bell et al., 2010). Common neurodegenerative diseases caused by blood–brain barrier disruption and subsequent NVU signaling interruption include AD, multiple sclerosis (MS), and stroke (Naranjo et al., 2022). Pericytes are also genetically associated with idiopathic basal ganglia calcification (IBGC). Sequencing of clinical samples revealed that p.Leu658Pro and p.Arg987Trp mutations in the pericyte-associated marker gene PDGFRβ as potential pathogenic mutations for IBGC (Nicolas et al., 2013). Here we will mainly use cerebral ischemia as an example to review and analyze the performance and function of brain pericytes under pathological conditions.

### 4.1. The epidemiology of ischemic stroke

Stroke is the second leading cause of death and disability, accounting for 5.2% of global death. In 2016, there were 13.7 million new cases of stroke and approximately 87% of them were ischemic stroke patients. Among them, at least 10–20% are due to occlusion of large arteries (Saini et al., 2021; Zhao et al., 2021). According to the report of the American Heart Association on heart diseases and stroke in 2020, the prevalence of stroke was 2.5%, equaling to 7 million people above the age of 20 having been inflicted by stroke. Among 800,000 stroke patients, nearly 150,000 died (Feske, 2021). Surprisingly, less than 5% of patients with ischemic stroke received intravenous thrombolysis within the time window. By 2016, less than 100,000 patients with ischemic stroke received such treatment (Saini et al., 2021). The BBB alteration characterized by degradation of junction proteins and increased permeability, especially after ischemic attack, is a key pathophysiological feature, which significantly impacts the treatment and prognosis of patients with ischemic stroke (Hu et al., 2017; Spitzer et al., 2022). It has been shown that injury to endothelial cells and pericytes of the NVU is the key factor that determines the progression and frequency of ischemic attack. Therefore, identification of types of cells, pathways, and molecules that influence the blood vessels may lead to new avenues to slow the progression of cerebrovascular diseases and reduce the frequency and impact of ischemic attack (Hu et al., 2017).

### 4.2. The performance and function of brain pericytes in ischemic stroke

In the CNS, vascular pericytes interact with endothelial cells to regulate the formation of tight junctions at the BBB. Under normoxic conditions, pericytes improve the endothelial integrity by inducing occludin and ZO-1 mRNA expression through angiogenin-1. Under hypoxic conditions, pericytes reverse the reduction in occludin protein expression and secrete GDNF which upregulates claudin-5 expression, thereby promoting brain or peripheral nerve regeneration (Wang et al., 2007; Shimizu et al., 2012; Faal et al., 2019).

Although pericytes accelerate barrier destruction in the short term at 1% O_2_ concentration, they have a positive effect after prolonged exposure and have more effective barrier protection function than astrocytes at 0.1% O_2_ concentration (Al Ahmad et al., 2009). Pericytes exhibit different effects at different stages of ischemia. At the initial stage when blood flow stops, matrix metalloproteinases (MMPs) in the cytoplasm of pericytes are rapidly activated promoting rapid hydrolysis and degradation of local proteins in the BBB, resulting in massive leakage of the capillary bed (Underly et al., 2017). Within the first 2 h of hypoxia, pericytes also begin to migrate with one out of every three pericytes migrating from their original position as confirmed in a study on cats (Gonul et al., 2002). All of these may prepare for subsequent angiogenesis.

Previous studies have shown that hypoxia is the main cause of angiogenesis. Firstly, hypoxia induces an increase in VEGF mRNA and protein in pericytes as atmospheric oxygen tension decreases with a corresponding increase in the expression of the fms-like tyrosine kinase 1 (flt1) gene which serves as the main form of VEGF receptor. Overexpression of VEGF not only promotes EC proliferation and budding but also promotes recruitment of pericytes by increasing n-cadherin expression on brain microvessels, enhancing endothelial cell coverage by pericytes thereby promoting formation and maturation of new blood vessels, increasing the density of capillaries and the blood flow in the infarct zone, reducing the infarct volume, mitigating inflammation after middle cerebral artery occlusion, enhancing integrity of the blood–brain barrier after ischemia, thereby improving brain tissue survival (Yamagishi et al., 1999; Zechariah et al., 2013; Shaikh et al., 2015). The TGF-β/TGF-βR2 and Ang/Tie2 pathways between pericytes and endothelial cells are major regulatory factors for vascular development. Hypoxia (2% O_2_ concentration) and VEGF (10 ng/mL) treatment significantly increase Ang1 mRNA expression, which reaches the maximum level 2 h after starting the treatment, followed by attenuation. The expression of angiogenin-2 (Ang2) mRNA does not change, but Tie2 mRNA is significantly upregulated, which persists for 12 h (Park et al., 2003; Sweeney et al., 2016).

Ischemia can also induce PDGFRβ(+) pericytes to express stem cell/undifferentiated cell markers and subsequently differentiate into various neural cells, including phagocytic microglia, thereby exerting neuroprotective effects (Nakagomi et al., 2015; Sakuma et al., 2016). The PDGFRβ-Akt signaling pathway is considered to be an important pathway for exerting neuroprotective effects after ischemic stroke. Studies have found that stroke induces an increase in PDGFRβ expression in pericytes and PDGF-BB expression in endothelial cells (Jung et al., 2011; Arimura et al., 2012). PDGF-BB significantly increases expression of the nerve growth factor (NGF) and the neurotrophic factor-3 (NT-3) in pericytes by inducing Akt phosphorylation in brain pericytes, thereby inducing cell growth and anti-apoptotic responses. In addition, it has been found that overexpression of the basic fibroblast growth factor (bFGF) significantly upregulates PDGFRβ expression in pericytes while 1% O_2_ and pH 6.5 acidification have no such effect. When the FGF receptor (FGFR) inhibitor SU5402 and PDGFR inhibitor Sunitinib are used, bFGF-induced pericyte proliferation and migration are significantly inhibited (Jung et al., 2011; Nakamura et al., 2016). Since pericytes in the CNS can promote neurogenesis and angiogenesis, they may mediate the repairing process of the neurovascular unit (Nakagomi et al., 2015).

Currently the widely used methods for treating ischemic stroke patients are intravenous thrombolysis and endovascular mechanical thrombectomy, but their application is still limited by time windows and related complications, such as edema, intracranial hemorrhage (Kim, 2019), which are related to the hypoxic pathological state of pericytes. In particular, exogenous tPA also severely damages pericytes leading to astrocyte detachment and reduced secretion of GDNF, disrupted NVU integrity (Deguchi et al., 2014). Even after upstream blood flow is restored microvascular perfusion remains reduced for a long time, a phenomenon known as “no reflow” which is attributed to persistent contraction of pericytes and their subsequent rigid death as this may irreversibly contract capillaries, impede red blood cell flow and disrupt the blood–brain barrier (Amarenco et al., 2009; Yemisci et al., 2009; Hall et al., 2014; Li et al., 2014; O'Farrell et al., 2017). Studies have found that a small increase in Ca^2+^ concentration in the cytoplasm of pericytes after stroke activates Ca^2+^ gated anion channel TMEM16A, promoting chloride ion efflux, depolarizing the cell membrane, opening voltage-gated calcium channels, thereby resulting in a large increase in intracellular calcium ions and pericyte contraction (Korte et al., 2022). Adenosine and nitric oxide also participate in pericyte contraction during ischemia–reperfusion (Caporarello et al., 2019). In addition, ROS produced by ischemia/ reperfusion activates specific secondary messenger pathways, leading to pericyte apoptosis and myosin translocation to the cytoskeleton, triggering the contraction mechanism of pericytes. However, 3-aminobenzamide and deferoxamine have been found to inhibit pericyte myosin translocation, contraction, and cell death (Shojaee et al., 1999).

In summary, the role of pericytes is two-sided after ischemic stroke. At the initial stage of disease onset and after blood flow recovery pericytes may cause persistent contraction due to changes in oxygen concentration or external stimuli, such as rtPA, exacerbating injury or blood–brain barrier disruption. In the late stage of disease onset, pericytes may differentiate into microglia and other neural cells through their stem cell activities to participate in the repairing process of the neurovascular unit. They may enhance communication with endothelial cells through their paracrine characteristics, promote angiogenesis, restore blood flow to the infarct zone and ultimately exert a positive effect.

## 5. Brain pericytes and neuroglial diseases

Glioblastoma (GBMs) is a highly lethal vascular brain tumor with rapid progression and frequent recurrence (Cheng et al., 2013; Zhang et al., 2021). Despite new radical surgical protocols, new cancer drugs, new treatment options, and advances in radiation technology, survival rates for GBMs have not improved significantly. The low efficacy of treatment and the short time interval between remission and recurrence can be attributed to the resistance of a small percentage of tumorigenic cells to treatment (Sharifzad et al., 2019). *In vivo* cell lineage tracing has shown that tumorigenic glioma stem cells (GSCs) are recruited by SDF-1/CXCR4 axial endothelial cells and induced primarily by TGFβ to become pericytes, which are typically involved in supporting vascular functions and tumor growth (Cheng et al., 2013). Studies have found that pericyte coverage of tumor vessels is negatively correlated with survival of GBM patients after chemotherapy (Zhou et al., 2017; Zhang et al., 2021). Therefore, selective elimination of GSCs-derived pericytes can destroy new blood vessels and effectively inhibit tumor growth (Cheng et al., 2013). Zhang et al. also found that pericytes control DNA damage repair (DDR) of GBM cells in the perivascular niche, thereby inducing temozolomide (TMZ) resistance (Zhang et al., 2021). Mechanistically, C-C motif chemokine ligand 5 (CCL5) secreted by pericytes activates C-C motif chemokine receptor 5 (CCR5) on GBM cells, enabling DDR mediated by DNA-dependent protein kinase catalytic subunits (DNA-PKcs) after TMZ treatment. Therefore, targeting CCL5-CCR5 signaling may be an effective therapeutic strategy and could improve the efficacy of chemotherapy for GBM. In addition, studies have found that high levels of LAMP2A in pericytes in GBM are also closely related to TMZ resistance and tumor progression (Auzmendi-Iriarte et al., 2022). Fibrotic scar tissue limits the regeneration of the central nervous system in adult mammals and is a pathological feature of many neurological diseases, such as ischemic stroke, multiple sclerosis, and glioblastoma. Fibrotic scarring and stromal cell distribution in human pathologies are specific to lesion types and, in most cases, are similar between the corresponding mouse models and humans. *In vivo* lineage tracing showed that in all mouse models that developed fibrotic tissue, the main source of scar forming fibroblasts was type a pericyte, a subtype of pericyte mainly found in the human brain and spinal cord. Therefore, treatment targeted on type a pericytes can be used as a therapeutic strategy to improve recovery after injury to the central nervous system (Dias et al., 2021).

## 6. Limitations and future directions

Pericytes play a crucial role in neurological diseases and they may be therapeutic targets for a variety of diseases, including stroke, glioblastoma, traumatic brain injury, migraine, epilepsy, spinal cord injury, diabetes, Huntington’s disease, AD, multiple sclerosis, glioma radiation necrosis, and ALS (Cheng et al., 2018). We believe that a deep understanding of the functional characteristics of the components of the NVU and their interactions is crucial for the treatment of ischemic stroke. However, most research has focused on endothelial cells, astrocytes, and microglia. Further exploration is needed to understand the mechanisms underlying pericyte’s functions. Pericytes appear to coordinate a number of key functions in stroke, including repair of the neurovascular unit, restoration of the blood flow and the permeability of the BBB (Liu et al., 2012). Ischemia and hypoxia triggers persistent contraction of pericytes leading to the “no reflow” phenomenon which severely worsens patients’ prognosis (O'Farrell et al., 2017). Therefore, development of drugs that can restore pericyte function and microvascular patency may improve the success rate of vascular reperfusion and neuroprotective therapies (Yemisci et al., 2009).

## Author contributions

JF: Visualization, Writing – original draft, Investigation, Methodology, Software. HL: Visualization, Writing – original draft, Writing – review & editing. PY: Data curation, Investigation, Visualization, Writing – review & editing. ZW: Investigation, Methodology, Software, Visualization, Writing – original draft. PZ: Conceptualization, Funding acquisition, Supervision, Writing – review & editing.

## Funding

The author(s) declare financial support was received for the research, authorship, and/or publication of this article. The present study was supported by a First-Class Construction Project of Medical Key Discipline of Yangpu District (YP19ZA08). The funder has no role in the design of the study and collection, analysis, and interpretation of data and in writing the manuscript.

## Conflict of interest

The authors declare that the research was conducted in the absence of any commercial or financial relationships that could be construed as a potential conflict of interest.

## Publisher’s note

All claims expressed in this article are solely those of the authors and do not necessarily represent those of their affiliated organizations, or those of the publisher, the editors and the reviewers. Any product that may be evaluated in this article, or claim that may be made by its manufacturer, is not guaranteed or endorsed by the publisher.
